# Biosynthesis of Organic Nanocomposite Using *Pistacia vera* L. Hull: An Efficient Antimicrobial Agent

**DOI:** 10.1155/2021/4105853

**Published:** 2021-07-09

**Authors:** Omolbanin Bakhshi, Ghodsieh Bagherzade, Pouya Ghamari kargar

**Affiliations:** Department of Chemistry, Faculty of Sciences, University of Birjand, Birjand 97175-615, Iran

## Abstract

Here presented a quick and easy synthesis of copper nanoparticles (CuNPs). Pistachio hull extract has been used as a reducing and stabilizing agent in the preparation of CuNPs. This biosynthesis is a kind of supporter of the environment because chemical agents were not used to making nanoparticles, and on the other hand, it prevents the release of pistachio waste in nature and its adverse effects on nature. The biosynthesized CuNPs and CuNPs/silver Schiff base nanocomposite (CSS NC) were characterized by UV-VIS spectroscopy, Fourier transform infrared spectroscopy (FT-IR), X-ray diffraction (XRD), field emission scanning electron microscopy (FE-SEM), and energy-dispersive X-ray spectroscopy (EDS). CuNP and CSS NC antimicrobial activity was examined by both well diffusion and determination MIC methods against four bacteria *Staphylococcus aureus*, *Bacillus cereus*, *Escherichia coli*, and *Pseudomonas aeruginosa* and two fungi *Aspergillus Niger* and *Candida albicans*. CuNPs and CSS NC showed significant antimicrobial activity on the samples, preventing the growth of bacteria and fungi at very low concentrations. CuNPs and CSS NC had the greatest effect on *Escherichia coli* bacteria and *Aspergillus niger* fungi. Phenolic compounds are one of the most important antioxidants that are involved in various fields, including pharmacy. *Pistacia vera* hull is a rich source of phenolic compounds. In this study, the most phenolic compound in *Pistacia vera* hull is gallic acid and rutin, which has been identified by HPLC analysis. In this study, *Pistacia vera* hull essential oil analysis was performed by the GC-MS method, in which *α*-pinene, D-limonene, and isobornyl acetate compounds constitute the highest percentage of *Pistacia vera* hull essential oil.

## 1. Introduction

Nanotechnology is the careful and controlled modification of the atomic or molecular structure of materials at the nanoscale to provide particles with new features and specific applications [1–4]. ‏Nanoparticles are particles having at least one dimension less than 100 nm [5, 6]. Particles at this scale have unique properties that, if properly constructed, can be applied in the fields of medical sciences, biotechnology and the environment, electronics, and energy [7–9]. Composites are materials that come from a combination of two or more constituents which have better properties than any of the materials alone [10, 11]. Composite materials are made of two components: one phase matrix component and the other component which is used as an amplifier. The composites are divided into three general categories: polymeric, ceramic, and metal based on the type of base material [12]. Now, if the nanoparticle is a component of the composite amplifier, it is called a nanocomposite. Nanocomposites have better performance than composites because of the unique properties of nanoparticles [13]. The use of nanoparticles as an antimicrobial in medicine is particularly important because their surface-to-volume ratios are high and therefore highly sensitive. Among metallic nanoparticles, copper metal has received considerable attention because of its low cost compared to gold and silver [14–16]. It seems that the mechanism of action of antibacterial effect of copper nanoparticles is the release of Cu^2+^ from these nanoparticles that due to having a positive charge, they absorb the negative charge of lipoproteins in the bacterial cell wall and enter the cell, damaging its cell wall or causing changes in its enzymatic function and causing holes in the cell wall. Overall, studies have shown that nanoparticles themselves can penetrate bacteria. This feature may be due to the induction of morphological changes in the cell wall as a result of which the material leaks out into the intracellular material and causes the death of bacteria [17]. Silver ions with dimensions of 1 to 10 nm have the most contact with the cell surface of bacteria, and their antibacterial effects are maximal. The exact mechanism of the effect of silver nanoparticles on microorganisms exactly is not clear, but the three mechanisms normally used by various researchers are as follows: (1) gradual release of silver ions resulting in inhibition of ATP production and DNA transcription, (2) direct damage to cell membranes by silver nanoparticles, and (3) generation of active oxygen radicals by silver nanoparticles and silver ions [18–20].

The nanoparticles stick together over time and the reaction progresses, thus reducing their efficiency and also making it difficult to recover from the reaction mixture. These problems can be solved by placing the nanoparticles on a suitable bed and producing nanocomposites [21]. Nanocomposite particles due to the high surface-to-volume ratio as well as a large fraction of the atoms on their surface increases the adhesion to the substrate are nanocomposites; that is, features are considered by many craftsmen to produce industrial coatings [22]. The particles that make up nanocomposites have dimensions smaller than most light wavelengths, so they have different absorption and radiance properties, which produce coatings that do not reduce the gloss and beauty of the surface and are transparent [23]. Nanocomposites have higher thermal, mechanical, and chemical resistance than other composites [24]. Nanocomposites often have high thermal and electrical conductivity [25]. Nanocomposites themselves are not refractory but can be used as an important component of a refractory complex [26]. Nanocomposites have a high barrier property, meaning that if used in packaging, they are a serious barrier to the passage of air, moisture, and other materials [24]. Classic methods of nanoparticle production include microwave, thermal decomposition, reverse micelles, electrochemical methods, etc. [27]. Most of these methods are expensive, and toxic materials are used in the working process [28]. As well as sometimes these reactions are possible only at high temperatures and pressures. To overcome these shortcomings, the scientists turned to the biological synthesis of nanoparticles. This method is compatible with the environment. It does not use toxins and does not produce toxins. Also, it achieves its goals with lower cost and higher efficiency [29].

**Scheme 1:**
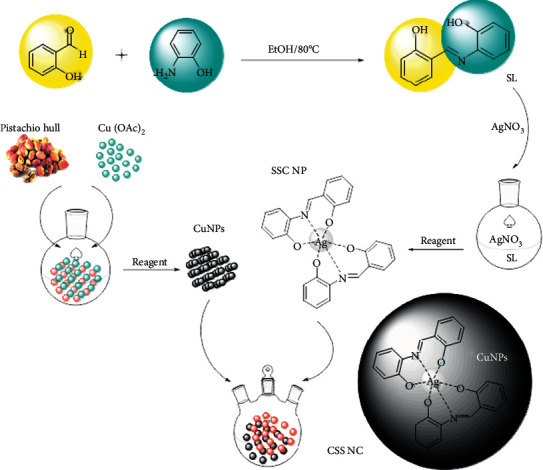
The synthesis procedure of CSS NC.

In this method, natural materials such as plants and micro-organisms are used to synthesize nanoparticles. Plants are a good choice because of their adaptability to the body and the environment [30]. In this method, plant extracts are used as both regenerative and stabilizing agents for the production of nanoparticles. In the nanoparticle green synthesis method, as opposed to the physical and chemical methods of nanoparticle synthesis, which include two steps of oxidation-resuscitation and stabilizer addition, there is a single step, and both regenerative and stabilizing substances are present in plant extracts resulting in energy consumption which will be affordable [31]. Pistachio belongs to the Anacardiaceae family of the *Pistacia* genus and *P*. *vera* species that grows in warm and dry areas [32]. Annually 30 million tons of pistachio waste is produced worldwide. The soft skin around the pistachio fruit contains many secondary compounds, and most of which are terpenes and phenolic compounds. Phenolic compounds include phenolic acids, anthocyanins, flavanols, isoflavones, flavanols 3-ol, flavanones, and flavones. Pistachio skin oil contains many compounds, and the main component of which is terpenes, such as limonene, *α*-pinene, camphene, caren, and others. The use of appropriate substrates for nanocomposite preparation enhances the sensitivity of nanocomposites [33]. Pistachio soft skin, because it has many biological polymers such as cellulose and tannic acid, has very good properties for use as a substrate in the preparation of nanocomposites, relatively high effective surface area, high chemical purity, high biocompatibility, good hydrophilicity, no need for harsh reaction conditions, nonadherence to nanoparticles, and reasonable price [34]. Due to the excessive use of antibiotics and increased resistance to bacteria, it is necessary to find suitable alternatives to antibiotics. For this reason, extensive studies have been conducted on the potential use of antimicrobial compounds in plants and the use of nanoparticles to control and treat pathogens [35]. Metal nanocomposite produced by biological methods has useful properties such as high level, small size, and high dispersion. The combination of these factors has led to a significant increase in the antimicrobial effects of metal nanocomposite compared to metals [36]. Due to the increasing antimicrobial effects of the metal nanocomposite, this material can be used to combat various pathogens, so that today with the development of nanotechnology, these nanocomposites have found many applications in various sciences such as medicine, pharmacy, cosmetics, and health [37]. The use of metal nanocomposites as a powerful bactericidal substance has flourished [38]. Metal nanocomposites can affect the metabolism and reproductive processes of microorganisms by inhibiting the respiratory system of bacteria and causing damage to the bacterial cell membrane [39]. Using pistachio peel to make nanocomposites in addition to the benefits mentioned above will not leave these wastes in nature. Due to the abundant presence of phenolic compounds in pistachio soft skin, if they are abandoned in nature, it will damage the environment and soil, including such as lowering soil pH, thereby reducing soil fertility and lowering groundwater quality [40]. The aim of this study was to analyze pistachio skin essential oil and identify a group of phenolic compounds in pistachio skin and synthesize nanoparticles and nanocomposites using pistachio waste with the least pollution and cost. The function of synthesized nanocomposite was investigated as antibacterial (Scheme 1).

## 2. Materials and Methods

All the reagents including copper (II) acetate monohydrate, silver nitrate, 2-aminophenol, and salicylic aldehyde were purchased from Merck and used without further refining. All the solutions were sterile. The Mueller–Hinton Broth culture medium was used to cultivate *Staphylococcus aureus*, *Bacillus cereus*, *Escherichia coli*, and *Pseudomonas aeruginosa* bacteria, and Sabouraud dextrose was used to cultivate the fungus such as *Aspergillus Niger* and *Candida albicans*. Gentamicin and clotrimazole antibiotics were used as positive controls. Fresh pistachios were harvested in September 2018 from Bardaskan, Razavi Khorasan, Iran.

### 2.1. Plant Collection and Preparation

Pistachio hull was collected in late August. Then, it was washed with cold purified water and dried completely in a cool, shady environment with proper ventilation. After ensuring that the moisture was completely removed, it was eaten by the mill and kept in a place without moisture and light for various tests.

### 2.2. Preparation of Pistachio Hull Extract (PHE)

In the first step, 15 grams of chopped dried pistachio skin was transferred to Erlenmeyer and 150 ml of deionized water was added to it. This extract for 20 minutes in an ultrasonic bath at a temperature of 45°C was sonicated and then centrifuged at 6000 rpm for 30 minutes to extract massive plant tissue is removed. Finally, the extract was filtered through a sinter funnel to remove particles of material and obtain a pure extract. The extract was stored in the refrigerator at 4°C for later stages.

### 2.3. Biosynthesis of CuNPs Using Pistachio Hull Extract

Add the above extract (8 mL) as a drop to solution of copper (II) acetate monohydrate (100 mL and 8 mM) during stirring and at 70°C water bath. In the first ten minutes, the color changed from light sky blue to yellow-green and then to olive green and then to dark olive. This mixing was continued for 5 hours, then the solution was centrifuged at 6000 rpm, and then the sediment was washed with distilled water and n-hexane and ethanol to remove uncoordinated phytochemical compounds. The blackish-brown powder obtained was dried overnight at room temperature and then dried for one hour in an oven at 65°C.

### 2.4. Antimicrobial Activity of CuNPs

**Figure 1:**
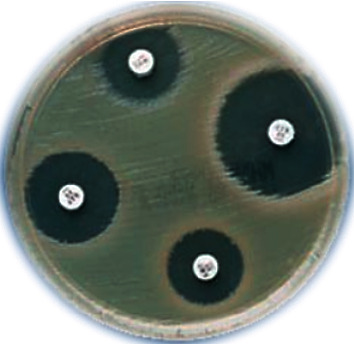
Aura of microbial nothing in the diffusion method from the well.

**Figure 2:**
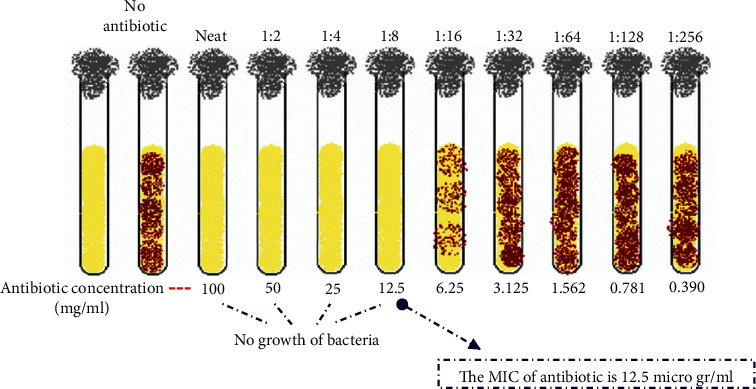
Schematic diagram of the method.

Microbial samples were regenerated using standard methods in Luria bertani and sabouraud dextrose media. To prevent the microbial suspension from 24-hour culture, each microorganism was inoculated separately into test tubes containing 3 mL of Mueller–Hinton Broths, and a suspension with turbidity equivalent to half McFarland was prepared. The antibacterial and antifungal effects of CuNPs were investigated by the well diffusion method in agar and MIC. In the method of agar well diffusion from the suspension of each microbe in the amount of 100 *µ*l on a plate containing Mueller Hinton agar for bacteria and Sabouraud dextrose agar for the fungi was poured and sterilized with sterile swap in three directions. Then, on the surface of each of the cultivated plates, wells with a diameter of approximately 6 mm and at a distance of 2 cm were created, and inside each well, 50 *µ*l was poured from each of the prepared CuNP dilutions with a sampler. The series of dilutions used to determine the aura of nongrowth in the propagation method from the well was 10, 20, 40, 60, and 100 micrograms per milliliter. And the antibacterial antibiotic gentamicin and the antifungal antibiotic clotrimazole were considered positive controls. After completing the work, the bacterial culture medium in the incubator was 37 degrees for 24 hours and the fungal cultures in the 28-degree incubator were incubated for 48 hours. Finally, after 24–48 hours, microbial cultures were evaluated for the formation or nonformation of nongrowth aura and the diameter of the formed auras was measured and reported in millimeters. To determine minimum inhibitory consent (MIC), the nanocomposite tubular dilution method was used. For this purpose, CuNPs prepared in test tubes containing 9 ml of the Mueller–Hinton Broth culture medium of series of dilutions of 1.56, 3.125, 6.25, 12.5, 25, 50, 100, and 200 mg.ml^−1^ were prepared. Then, give each tube 1 ml of suspension, and prepared microbe was inoculated. The CuNPs were used as a positive control. For negative control, the suspension of CuNPs and culture medium were placed in another tube. Cultivation containing a solution with a concentration of half a microgram per milliliter of CuNPs was added. Finally, all test tubes were transferred to incubators at 37°C for bacteria and 28°C for fungi for 24–48 hours. After incubation, each tube was examined for turbidity due to the growth of the microorganism and the lowest value in which the turbidity was not due to the inhibitory effect of nanocomposite was considered as MIC (Figures 1 and 2).

### 2.5. Synthesis of (E)-2-((2-Hydroxybenzylidene) Amino) Phenol Schiff Base Ligand (SL)

**Scheme 2:**
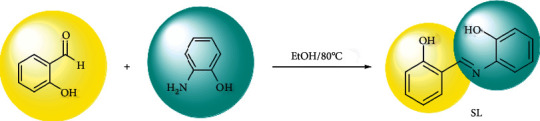
Chemical reaction of Schiff base synthesis.

The solution of (1 mmol) 2-aminophenol in 5 ml EtOH was added dropwise to the solution of salicylaldehyde (1 mmol) in 5 ml EtOH. The mixture was refluxed for 2 h. After the finished reaction, the solution was centrifuged at 6000 rpm to the orange precipitate, separated faster than the solution. This precipitate was washed with reaction solvent (3 × 10) and then dried in a vacuum. The synthesis of SL is shown in Scheme 2.

 
^**1**^**HNMR** (DMSO, 300 MHz): *δ* = 6.61–7.72 (m, 7H, H-Ar), 8.33 (s, 1H, CH=N), 9.41 (s, 1H, OH of 2-aminophenol), and 10.64 (s, 1H, OH of salicylaldehyde) ppm.

### 2.6. Synthesis of Silver Schiff Base Complex (CSS)

**Scheme 3:**
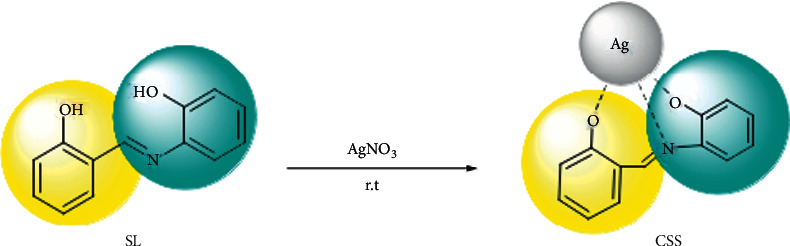
Chemical reaction of Schiff base complex synthesis.

An ethanol solution of silver nitrate (5 mL, 0.2 M) dropwise to ethanol solution of SL (10 mL, 0.1 M) was added during sonicated for 15 minutes. The color of the solution changed from orange to blood red. The resulting solution was stirred at room temperature for 2 hours on a stirrer. After 2 hours, the solution was centrifuged and a silver-colored precipitate was obtained, which was washed three times, each time with 10 cc ethanol. The precipitate was then dried at room temperature. The synthesis of CSS is shown in Scheme 3.

 
^**1**^**HNMR** (DMSO, 300 MHz): *δ* = 6.64–7.78 (m, 7H, H-Ar) and 8.43 (s, 1H, CH=N) ppm.

### 2.7. Synthesis of CuNPs/Silver Schiff Base Nanocomposite (CSS NC)

**Scheme 4:**
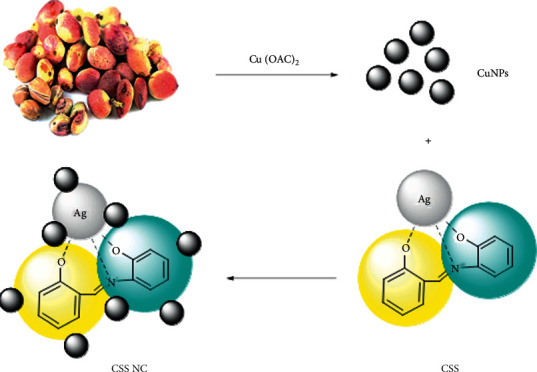
Chemical reaction of CSS NC synthesis.

Then, the aqueous solution of CSS (800 *µ*L, 0.01 M) was added to CuNPs (0.08 g) in water (10 mL) during stirring for an hour and then centrifuged for 20 minutes. The synthesis of CSS NC is shown in Scheme 4.

### 2.8. Antimicrobial Activity of CSS NC

Microbial samples were regenerated by standard methods in Luria Bertani and Sabouraud dextrose media. To prevent the microbial suspension from 24-hour culture, each microorganism was inoculated separately into test tubes containing 3 mL of Mueller–Hinton Broths, and a suspension with turbidity equivalent to half McFarland was prepared. The antibacterial and antifungal effects of CSS NC were investigated by the well diffusion method in agar and MIC. In the method of agar well diffusion from the suspension of each microbe, the amount of 100 *µ*l on a plate containing Mueller Hinton agar for bacteria and Sabouraud dextrose agar for the fungi was poured and sterilized with sterile swap in three directions. Then, on the surface of each of the cultivated plates, wells with a diameter of approximately 6 mm and at a distance of 2 cm were created, and inside each well, 50 *µ*l was poured from each of the prepared CSS NC dilutions with a sampler. The series of dilutions used to determine the aura of nongrowth in the propagation method from the well was 10, 20, 40, 60, and 100 micrograms per milliliter, and the antibacterial antibiotic gentamicin and the antifungal antibiotic clotrimazole were considered positive controls. After completing the work, the bacterial culture medium in the incubator was 37 degrees for 24 hours and the fungal cultures in the 28-degree incubator were incubated for 48 hours. Finally, after 24–48 hours, microbial cultures were evaluated for the formation or nonformation of nongrowth aura and the diameter of the formed auras was measured and reported in millimeters. To determine minimum inhibitory consent (MIC), the nanocomposite tubular dilution method was used. For this purpose, CSS NC prepared in test tubes containing 9 ml of the Mueller–Hinton Broths culture medium of series of dilutions of 1.56, 3.125, 6.25, 12.5, 25, 50, 100, and 200 mg.ml^−1^ was prepared. Then, give each tube 1 ml of suspension, and prepared microbe was inoculated. The CSS NC was used as a positive and negative control of the tube containing the culture medium with a microbe, to ensure that the work steps were sterile, as well as the CSS NC suspension, inside one tube and in another tube and the culture medium in another tube. Cultivation containing a solution with a concentration of half a microgram per milliliter of CSS NC was added. Finally, all test tubes were transferred to incubators at 37°C for bacteria and 28°C for fungi for 24–48 hours. After incubation, each tube was examined for turbidity due to the growth of the microorganism and the lowest value in which the turbidity was not due to the inhibitory effect of nanocomposite was considered as MIC.

#### 2.8.1. Extraction of Pistachio Skin Methanolic Extract to Identify Phenolic Compounds

For this purpose, 15 g of the dried pistachio skin sample was transferred to an Erlenmeyer flask, 100 ml of distilled methanol solvent was added to it, and it was stirred on the shaker for 72 hours at room temperature. Extraction was performed. The extract was filtered and then analyzed by HPLC.

#### 2.8.2. Isolation of Essential Oil

The completely dried pistachio hull powder was placed in Clevenger with deionized water for 6 hours. In this way, pistachio skin oil was obtained. The oil was then dehumidified with sodium sulfate, and its GC-MS was taken. GC-MS results qualitatively identify pistachio skin oil components.

#### 2.8.3. Statistical Tools

GC/MS analysis of the oil was carried out on an Agilent HP-6890 gas chromatograph. A Waters liquid chromatography apparatus consisting of a separation module, Waters 2695 (USA), and a PDA Detector Waters 996 (USA) was used for the HPLC analysis. Data acquisition and integration was performed with Millennium32 software. The chromatographic assay was performed on a 15 cm × 4.6 mm, with precolumn, Eurospher 100-5 C18 analytical column provided by Waters (SunFire) and reversed phase matrix (3.5 *μ*m) (Waters), and elution was carried out in a gradient system with methanol as the organic phase (solvent A) and distilled water (solvent B) with the flow-rate of 1 mL min^−1^. Peaks were monitored at 195–400 nm wavelength. Injection volume was 20 *µ*L, and the temperature was maintained at 25°C. UV-Vis spectra were recorded using a Shimadzu UV-Vis 160, a double-beam spectrophotometer. FT-IR spectra were obtained using a JASCO FT-IR 4600 spectrophotometer using KBr pellet. X-ray diffraction (XRD) measurements were performed with the Philips PW1730 model device, and the scanning rate was 0.5 ^0^ min^−1^ within the 2*θ* range of 10^0^ to 80^0^. The size and shape of the nanoparticles were identified by scanning electron microscope (SEM) using FE-SEM TESCAN MIRA3 that equipped with BDT technology to create excellent resolution at low voltage. To determine the morphology and size of nanoparticles, TEM analysis with accelerator voltage 100 kV and maximum resolution less than 0.2 nm was used. To determine the morphology and size of nanoparticles with Philips EM208S 100 KV with accelerator voltage 100 kV and maximum resolution less than 0.2 nm, TEM analysis was performed. Imaging was performed in two modes: Bright Field and Dark Field. The constituent elements of nanocomposites and their weight percentage were explored on a field-emission-scanning electron microscope (FE-SEM) equipped with an energy dispersive X-ray spectrometer (EDS) (Cam scan MV2300).

## 3. Results and Discussion

There are two basic points in nanoparticle production: existence of reducing compounds and the presence of stabilizing compounds that prevent the nanoparticles sticking together and clumping. Due pistachio skin having large amounts of phenolic compounds can be used as a reducing agent to reduce metal ions in the synthesis of metal nanoparticles. In addition to its regenerative role, pistachio skin produces a coating around the nanoparticles, which prevents the nanoparticles from clotting and sticking together. In this study, first, CuNPs were synthesized using pistachio skin. The nanoparticles stick to each other over time, which reduces the efficiency of the nanoparticles and makes it difficult to recycle the nanoparticles from the reaction solution. Therefore, to synthesize CSS NC, we placed Cu nanoparticles on a CSS as a complex.

### 3.1. Synthesis of CuNPs

**Figure 3:**
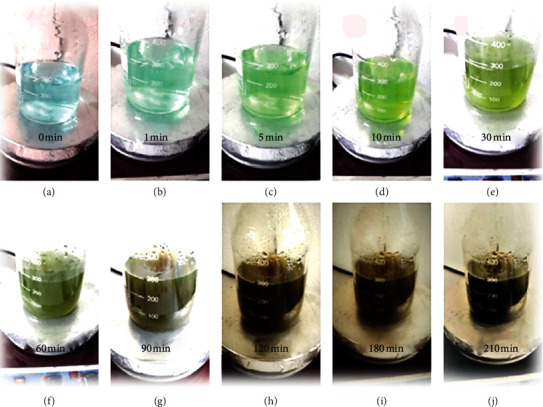
The color change of the biosynthesized CuNPs using pistachio hull extract in varying time intervals.

First to identify CuNPs, various spectroscopic methods such as UV-Vis, XRD, and SEM were used. UV-Vis spectroscopy is the most widely used technique for structurally describing nanoparticles. Changing the color of the solution from light sky blue to yellow-green and then to olive green and then to dark Olive Maya to black was a reason to prove the formation of CuNPs (Figure 3). This color change is caused by the excitation of the surface plasmon vibrations of CuNPs. The surface plasmon resonance of CuNPs produces a peak of about 500 nm [41].

**Figure 4:**
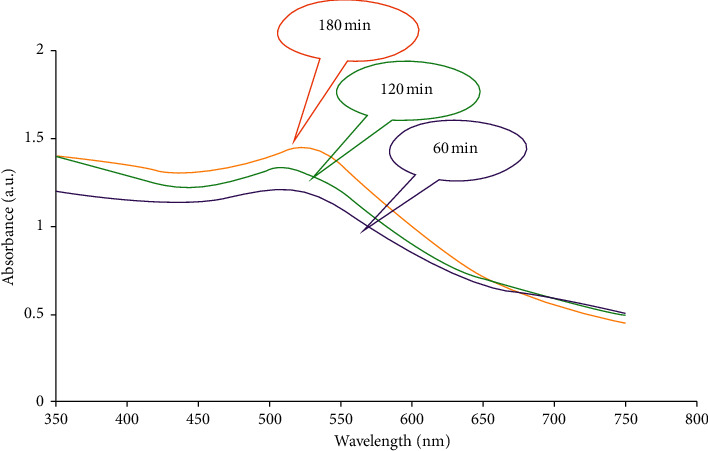
UV-Vis absorption spectrum of CuNP biosynthesis at different time intervals.

The role of time is one of the important parameters in the process of reacting and forming nanoparticles. As shown in (Figure 4), over time the intensity of the surface plasmon resonance peak increases in the range of 500 nm. Surface plasmon resonance is a feature of metal nanoparticles with sizes of 2–100 nm. Therefore, when the surface plasmon resonance intensifies over time, it means that the synthesis of CuNPs with a size of 2–100 nm has increased.

**Figure 5:**
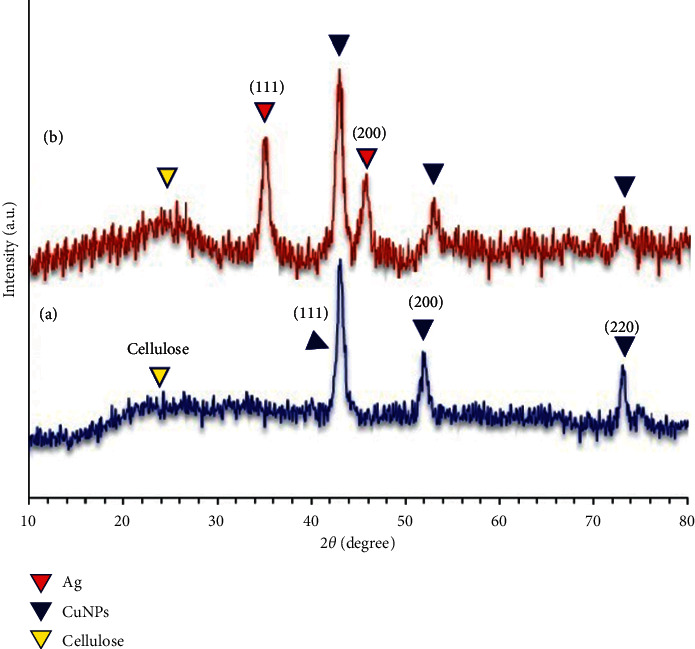
XRD pattern of the biosynthesized CuNPs: (a) using pistachio hull extract and CSS NC (b).

The X-ray diffraction patterns of the CuNPs and CSS NC are shown in Figure 5. As shown in Figure 5(a), it can be seen that the characteristic broad diffraction peaks shown in wide-angle XRD patterns can be indexed to the CuNP structure, which shows diffraction peaks, and the patterns indicate a crystallized structure at 2*θ*: 44.5°, 52.8°, and 73.6° which are assigned to the (111), (200), and (220) and with reference card (no .71e4610) corresponds to [42]. Since no scattering of copper oxide (I) and copper oxide (II) is observed, the successful synthesis of CuNPs is confirmed. XRD pattern in Figure 5(b) shows that peaks at 2*θ*: 35.6° and 45.9° correspond to the (111) and (200) crystallographic phases in XRD pattern that are related to Ag [43]. As a result, the XRD pattern of CSS NC is shown in Figure 5(b) and the XRD pattern shows the characteristic peaks of bare Ag and Cu NPs at 2*θ*=10–80 corresponding to the reflections of the (111), (111), (200), (200), and (220) planes. While cellulose is the main component of pistachio skin, in which the peak appeared at 2*θ* = 20.3–24.2° which attributed to the cellulose species [44].

**Figure 6:**
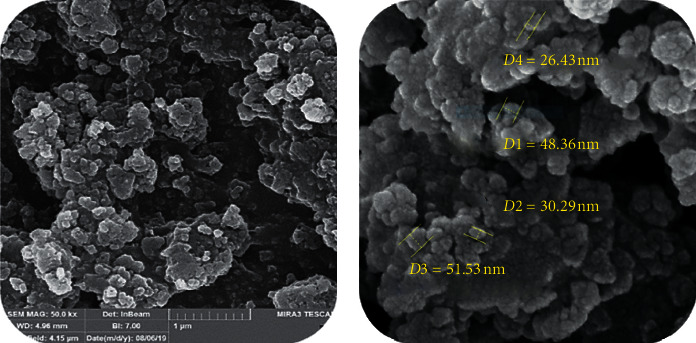
SEM image of the biosynthesized CuNPs by using pistachio hull extract.

SEM is used to determine the size of CuNPs. The SEM image of CuNPs is shown in Figure 6. According to this image, the diameter of the CuNPs is in the range of 26–51 nm.

**Figure 7:**
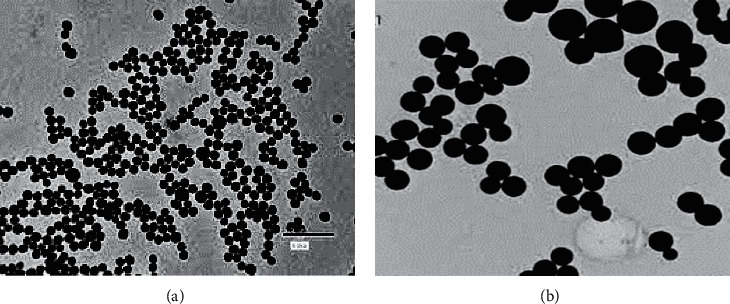
TEM images of CuNPs.

The TEM image of CuNPs is shown in Figure 7. Spherical morphology with low tendency to accumulation and distribution with very narrow diameter is observed. According to this image, the diameter of CuNPs is 15–45 nm.

### 3.2. Review and Comparison of PHE and CuNPs

**Figure 8:**
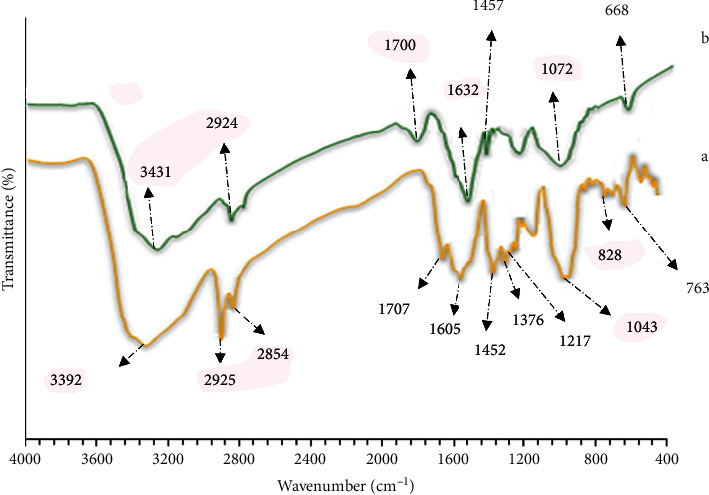
FT-IR of PHE (a) and FT-IR of CuNPs (b).

The IR spectrum of PHE and CuNPs is shown in Figure 8. Figure 8(a) represents the FT-IR spectrum of PHE. It displays major vibrational absorptions at 3492 (broadband), 1707, 1605 and 1452, 1376, 1217, 1043, 828, and 763 cm^−1^ which are ascribed to phenolic O-H stretching, carbonyl group stretching, C=C aromatic ring stretching, methylene C-H bending, O-H bending, C-O stretching, aromatic C-H out of plane bending, and O-H out of plane bending vibrations, respectively. The observed absorption bands at 2854 and 2925 cm^−1^ are attributed to the C-H asymmetric and symmetric stretching bands of the alkyl chains present in the structure of the pistachio hull components [45, 46]. The FT-IR spectrum of the prepared CuNPs (Figure 8(b)) was recorded to identify the functional groups of the capping and stabilizing agents present on their surface. As can be seen, this spectrum is very similar to that of powdery PHE (Figure 8(a)), indicating that the CuNPs are stabilized via surrounding by at least the major constituents of PHE. However, some minute differences can be detected between the two spectra. In the FT-IR of the CuNPs, the O–H bond shifted to a lower wavenumber and appears at 3341 cm^−1^, which illustrates the formation of Cu nanoparticles in PHE. This blue shift implies that the PHE capping layer of the CuNPs is very thin, so the hydroxyl groups of this layer exist majorly on surface of the nanoparticles and are not involved in intermolecular H-bonding as extensive as they experience in the bulk of PHE. This seems to be also the case for the O-H bending absorption band, in which its peak shifts from 1605 cm^−1^ for pure PHE to around 1620 cm^−1^ for the PHE on surface of the CuNPs. Presumably, most of the organic compounds covering the surface of the CuNPs are the residue of the PHE already coordinated with Cu^2+^ during its reduction [41]. In addition, this band is broad, signifying that the generated CuNPs are crystalline in nature [47]. There is also a weak band in this spectrum at 668 cm^−1^ which arises from Cu-O stretching vibration. These FT-IR data prove that the phytochemicals of PHE have strong ability to bind with the metallic Cu and prevent CuNPs from agglomeration. Certainly, the biological molecules of PHE play the key roles of reducing, stabilizing, and capping agents in the aqueous production of CuNPs.

### 3.3. Antimicrobial Activity (CuNPs)

Due to the growing prevalence of infectious diseases and food poisoning caused by microbes due to the development of resistant strains of various antibiotics in these microbes, researchers thought of using new antimicrobials against microbes. Usually, green nanoparticles synthesized have better antimicrobial and antifungal activity against microorganisms. Hence, more attention has been paid to the synthesis of antimicrobial nanoparticle [48]. The results of CuNP antimicrobial tests by the well diffusion method and MIC determination are presented in Tables 1 and 2, respectively. CuNPs has a significant microbial effect on case studies, and experiments showed that at very low concentrations, it prevented the growth of bacteria and fungi. The results show that CuNPs have the greatest effect on *Escherichia coli* and *Aspergillus niger* and had the least effect on *Staphylococcus aureus*.

#### 3.3.1. Synthesis of SL and CSS

**Figure 9:**
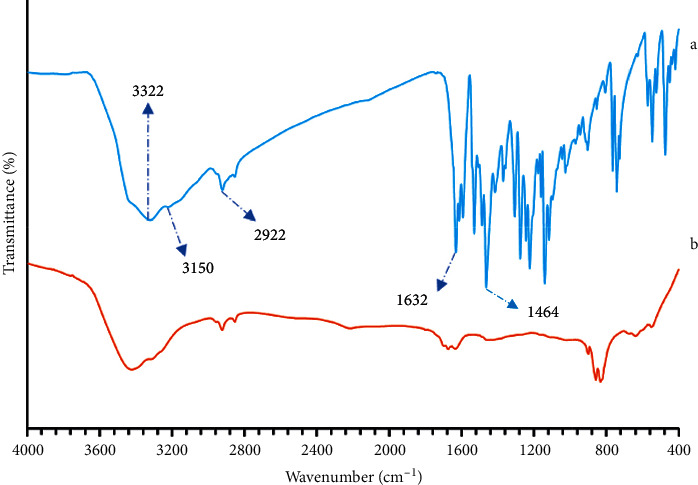
FT-IR of SL (a) and SCC (b).

The FT-IR method was used to identify SL and CSS. The FT-IR spectra of SL 9(a) and CSS 9(b) are shown in Figure 9. In the SL spectrum, the peak observed at 3322 cm^−1^ corresponds to the stretching vibrations of O-H, and the peak observed at 1632 cm^−1^ indicates the double bond of carbon and hydrogen in SL structure. The absorption peak at 1464 cm^−1^ is related to aromatic C=C, and the absorption peak in 3150 cm^−1^ is related to aromatic C-H in the structure of the benzene ring in SL. In the SL spectrum, the absorption peak at 2922 cm^−1^ is related to aliphatic C-H [49]. As can be seen in the CSS spectrum, peaks have disappeared due to the interaction of oxygen and nitrogen in SL and the silver metal.

#### 3.3.2. Synthesis of CSS NC

**Figure 10:**
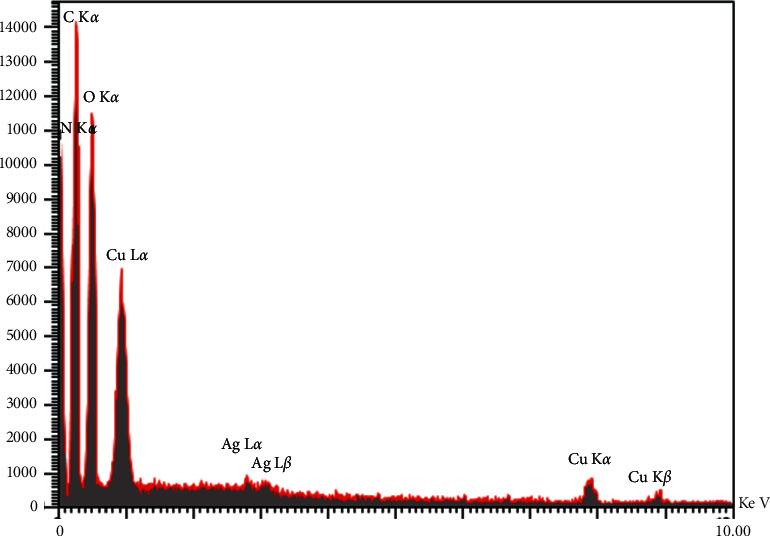
The EDS spectrum of CSS NC.

EDS analysis was used to identify the constituent elements of CSS NC, and the results are shown in Figure 10.

#### 3.3.3. Antimicrobial Activity (CSS NC)

The results of CSS NC antimicrobial tests by the well diffusion method and MIC determination are presented in Tables 3 and 4, respectively. CSS NC has a significant microbial effect on case studies, and experiments showed that at very low concentrations, it prevented the growth of bacteria and fungi. The results show that CSS NC has the greatest effect on *Escherichia coli* and *Aspergillus fungi* and had the least effect on *Staphylococcus aureus*.

Comparing Tables 1 and 3, it can be seen that the CSS NC has increased the diameter of the growth inhibition zone of microorganisms in all species of microorganisms in comparison with CuNPs. Comparing Tables 2 and 4, it can be seen that CSS NC prevents the growth of microorganisms in concentrations lower than CuNPs, and this is true for all microorganisms. Therefore, in general, CSS NC has more antimicrobial effects than CuNPs.

### 3.4. HPLC Analysis

Phenolic compounds are the most important antioxidants. The ability to use phenolic-based drugs is to prevent or treat some diseases that are caused by free radicals. These include cancer, atherosclerosis, ischemia, neuronal degenerative diseases, and cardiovascular disease [50]. The results of measuring the phenolic compounds of pistachio skin methanolic extract using HPLC analysis are given in Table 5. Also, we show the structure of phenolic compounds identified in *Pistacia vera* L. hull in Table 6.

Free radicals (active oxygen) are powerful oxidizing agents in the body that are known to cause a variety of diseases, aging, and wrinkles. Free radicals are increased by overexposure to UV rays, aging, and smoking. Phenolic compounds are one of the most important sources of antioxidants [51].  Table 7 compares the amount of phenolic compounds in *Pistacia vera* L. hull in this study and similar research.

### 3.5. Investigation of Medicinal Properties of Pistachio Skin Phenolic Compounds

In the following, we will briefly introduce and review the medicinal properties of each of the known phenolic compounds in *Pistacia vera* L. hull. The name of the gallic acid is trihydroxybenzoic acid. According to this research, gallic acid forms a significant part of pistachio skin. Gallic acid has antifungal and antibacterial properties. Gallic acid acts as an antioxidant and helps the body's cells protect against oxidative damage. Gallic acid has been shown to have anticancer properties without damaging the body's cells. It is also used in internal bleeding. It is also used as a medicine in the treatment of albuminemia and diabetes. Gallic acid ointments are used as an ointment in the treatment of psoriasis and haemorrhoidal bleeding [53]. Rutin is a natural pigment that has many applications in the food industry, including preservation and stabilization. This compound has a variety of bioactive activities including antioxidant, anti-inflammatory, and protective effects on the liver that are beneficial to human health [54].

Rutin can help maintain the elasticity of blood vessels, reduce the fragility of blood vessels, reduce capillary permeability, and prevent the effects of high blood pressure. This antioxidant can inhibit acute and chronic inflammation. This is a potential natural cure for arthritis. Rutin is able to reduce the production of free radicals in rheumatoid arthritis. Routine can act as a therapeutic agent in the fight against cancer due to its antioxidant and anti-inflammatory effects. It also stimulates apoptosis or cancer cell death. This flavonoid has been shown to have anticancer effects against a number of cancers, including leukemia, colon cancer, colorectal cancer, liver cancer, and lung cancer. Rutin can protect us from the growth of metabolic syndrome. Research suggests protective effects on brain damage and age-related injuries. It also helps improve brain health due to its anti-inflammatory and antioxidant properties‏ [55]. This bioflavonoid acts as a promising compound for the treatment of neurodegenerative diseases, including Alzheimer's disease, Parkinson's disease, and Huntington's disease. Researchers believe that by maintaining the cytokines of inflammatory proteins, improving the enzymatic activity of antioxidants, and restoring the activity of complex mitochondrial enzymes in our cells, it maintains brain health. Rutin inhibits protein disulfide isomerase (PDI), which is rapidly secreted by platelets and endothelial cells during thrombosis. Thrombosis is when a blood clot forms in a vein or artery. Rutin not only inhibits PDI but also prevents it from entering cells. The researchers found that this helps prevent blood clots from forming in the arteries and veins. Rutin is used to reduce varicose veins, eliminate hemorrhoids, and prevent hemorrhagic strokes caused by broken blood vessels or arteries. Rutin significantly reduces foot swelling, foot pain and leg cramps, heaviness, and itching. This is probably due to its ability to reduce inflammation and improve blood circulation [56]. Catechin is a flavan-3-ol, a type of natural phenol and antioxidant. It belongs to the group of flavan-3-ols, part of the chemical family of flavonoids. Excess LDL cholesterol can cause oxidation of cells in the body and cause atherosclerosis. Catechin limits bad cholesterol (LDL) and is therefore helpful in preventing atherosclerosis. Catechin not only lowers bad cholesterol (LDL) but also raises the level of good cholesterol (HDL) in the body. Many studies have shown that eating catechins increases healthy energy consumption in humans and animals and reduces body fat and the digestive system. Catechin absorbed into the bloodstream has also been reported to be transported to liver cells and help increase fat metabolism [57]. Catechin has strong antibacterial and sterilizing effects on germs and bacteria. In this way, catechin is also useful to prevent infection and disease [58]. ‏Catechin has a moderating effect on allergies (including airborne dust allergies). Catechin reduces allergy symptoms including itching and lenses. Catechin protects your enamel by reducing harmful acids in the mouth that can destroy tooth enamel [59]. Quercetin is a type of polyphenol and flavonoids. Quercetin has a number of anti-inflammatory properties in the same way that aspirin can reduce inflammation and related pain. This causes the substance to block the chemical pathway of some of the ways in which inflammation is produced. As a result, quercetin is able to reduce pain from arthritis, gout, rheumatism, and general inflammation, in which people usually suffer from colds, fevers, muscle aches, and high blood pressure [60]. ‏It has been shown to have different aldose reductase inhibitory properties. Some of the problems that occur in diabetics are due to the sorbitol pathway in which high concentrations of glucose are converted to fructose and sorbitol. This can lead to glaucoma, cataracts and various neurological complications. Quercetin is an aldose reductase inhibitor, so it prevents this huge change and helps manage the problems of patients with diabetes [61].‏ The bioflavonoids reduce the amount of chemicals involved in inflammatory reactions or allergens in the body. Thus, quercetin can reduce the severity of asthma attacks that respond to these chemicals and reduce various forms of congestion that are not associated with asthma [62]. ‏Quercetin has been linked to a reduction in pancreatic, prostate, colorectal, and skin cancers [63]. Quercetin has the ability to lower blood pressure and eliminate the effects of this disease [64]. As an anticoagulant, anti-inflammatory, and antioxidant, quercetin helps keep your heart healthy. Quercetin is regularly used in allergy treatments because antioxidants with quercetin-like structures reduce the amount of histamine released when stimulating an allergic pathway. Decreased histamines mean reduced symptoms, and quercetin intake is particularly associated with reduced symptoms of eczema, urticaria, and hay fever [62]. Protocatechuic acid (PCA) is anti-inflammatory and antioxidant. PCA protected against chemically induced liver toxicity in vivo. In vitro testing documented anti-inflammatory and antioxidant activity of PCA, while liver protection *in vivo* was measured by histological assessment and chemical markers. [65] PCA has been reported to induce apoptosis of human leukemia cells [66]. PCA increases proliferation and inhibits neuronal stem cell apoptosis [67]. PCA showed an excellent ability to effectively inhibit the replication of herpes simplex virus type 2 [68]. Essential oils are chemical and aromatic compounds found in various plants, of which about 700 species have been identified to date. Essential oils are naturally occurring in a wide variety of compounds, such as terpenoid alcohols, hydrocarbons, phenols, aldehydes, esters, and ketones. Often one or more of these compounds affect the aroma of the essential oil. These plants and their essential oils cover a wide range of uses. Their constituent compounds can have medicinal properties and be included in the formulation of drugs. Their scent is used in the perfume industry, fragrances, and a type of therapy (aromatherapy). The flavoring properties of essential oils are widely used in a wide range of foods, toothpastes, mouthwashes, beverages, and even tobacco. Its saturated vapors have the ability to repel pests and insects and are used in some industrial pesticides [69].

### 3.6. GC-MS Analysis

**Figure 11:**
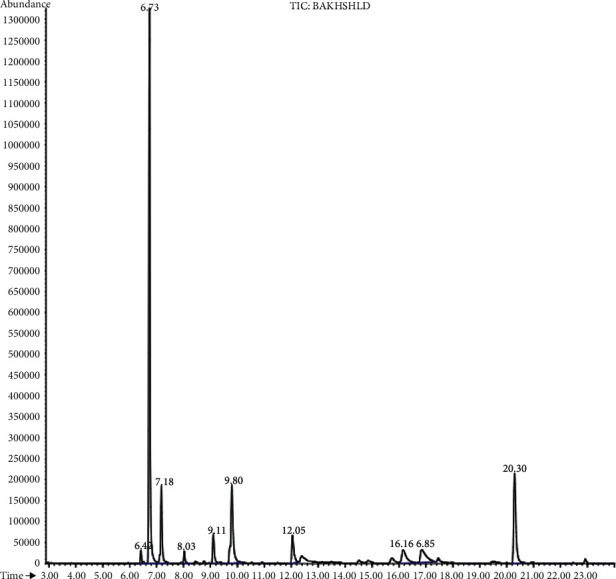
GC-MS analysis of *p. vera* methanolic extract.

GC-MS analysis for the *P. vera* methanolic extract is shown in Figure 11. In this study, eleven compounds were identified in methanolic extract.

The chemical composition of the essential oil (EO), which was identified by GC-MS analysis, is shown in Table 8.

Among the compounds identified as tricyclene, *α*-pinene, camphene, *β*-pinene, 3-caren, D-limonene, terpinolene, limonene-4-ol, *α*-terpineol, and isobornyl acetate are terpens. Terpenes are compounds that result from the “head-to-tail” connection of two or more isoprene units. The structure of the identified terpenes is shown in Table 9.

*α*-Pinene, D-limonene, and isobornyl acetate had a higher percentage than other terpenes, which we will briefly introduce each of them below. *α*-Pinene is a colorless liquid and one of the two isomers of pinene. *α*-Pinene is an alkene and contains a 4-membered reaction ring. It has antimicrobial and anti-inflammatory properties and helps memory as an acetylcholinesterase inhibitor [70]. D-Limonene is a colorless liquid monoterpene with the chemical formula C_10_H_16_. It is used as a flavoring in food and as a flavoring in perfumes and cosmetics, used in detergents to clean glue and oil. Other applications include its use as a plant insecticide. D-Limonene may irritate the skin if used directly on the skin. D-Limonene is toxic to aquatic life [71]. Isobornyl acetate is a colorless liquid monoterpene with the formula C_12_H_20_O_2_. Due to its pleasant smell, it is used in cosmetics and perfumes. In high doses, it may irritate the skin. Frequent and excessive exposure to isobornyl acetate may cause kidney and liver damage [72].

## 4. Conclusions

Pistachio peel is a rich source of phenolic compounds such as gallic acid, protocatechuic acid, and rutin. The mentioned compounds, due to having electronic sources, are suitable agents to reduce metal salts and convert them into metal particles. They act as stabilizers for synthesized nanoparticles; hence, pistachio peel extract is an alternative option for synthesizing nanoparticles (CuNP) with a size between 51 and 51 nm. In addition, strict laboratory conditions and toxic compounds are not used during the synthesis process. CuNPs are placed on the CSS substrate to prevent their mobility and accumulation, while their effectiveness is not reduced. On the other hand, it is possible to easily recover them from the reaction solution. This nanoparticle (CuNP) and nanocomposites (CSS NC) have biological properties such as antibacterial and antifungal. The experiment was performed on 4 bacteria (*Staphylococcus aureus*, *Bacillus cereus*, *Escherichia coli*, and *Pseudomonas aeruginosa*) and two fungi (*Aspergillus niger* and *Candida albicans*). The results show the successful role of nanoparticles and nanocomposites made using pistachio skin extract. Considering the important role of terpene in plants and community health, it was decided to study the structure of *Pistacia vera* hull oil. The essential oil of the *Pistacia vera* hull was isolated using Clevenger, and then its terpenes were identified using a device GC-Mass. According to the results, the highest percentage of compounds is related to terpenes such as D-limonene, *α*- pinene, and ester isobornyl acetate because the annual production of pistachios in Iran is a significant figure, so the nanoparticles and nanocomposites created can be considered as an effective candidate in pharmacy and medicine.

## Figures and Tables

**Table 1:** Nonmicrobial growth diameter at various CuNP concentrations by diffusion from wells (in millimeters).

Microorganism	Different concentrations of CuNPs (*µ*g.ml^−1^)						
	10	20	40	60	100	Gentamicin (50 *µ*g/ml)	Clotrimazole (50 *µ*g/ml)
*Staphylococcus aureus*	0	7	12	14	17	25	—
*Bacillus cereus*	7	9	11	14	18	24	—
*Escherichia coli*	9	11	14	17	22	23	—
*Pseudomonas aeruginosa*	8	11	13	17	20	25	—
*Aspergillus niger*	9	12	15	18	21	—	26
*Candida albicans*	9	11	14	17	20	—	25

**Table 2:** The minimum inhibitory concentration (MIC) of microbe's CuNPs in different concentrations (measured in *µ*g.ml^−1^).

Microorganism	Different concentrations of CuNPs (*µ*g.ml^−1^)									
	0.78	1.56	3.12	6.25	12.5	25	50	100	200	400
*Staphylococcus aureus*	+	+	+	+	+	−	−	−	−	−
*Bacillus cereus*	+	+	+	+	−	−	−	−	−	−
*Escherichia coli*	+	+	−	−	−	−	−	−	−	−
*Pseudomonas aeruginosa*	+	+	+	−	−	−	−	−	−	−
*Aspergillus niger*	+	+	−	−	−	−	−	−	−	−
*Candida albicans*	+	+	+	−	−	−	−	−	−	−

**Table 3:** Nonmicrobial growth diameter at various CSS NS concentrations by diffusion from wells (in millimeters).

Microorganism	Different concentrations of CSS NC (*µ*g.ml^−1^)						
	10	20	40	60	100	Gentamicin (50 *µ*g/ml)	Clotrimazole (50 *µ*g/ml)
*Staphylococcus aureus*	1	9	14	17	21	25	—
*Bacillus cereus*	8	11	13	17	22	24	—
*Escherichia coli*	10	13	16	20	26	23	—
*Pseudomonas aeruginosa*	9	13	15	20	24	25	—
*Aspergillus niger*	10	14	17	21	25	—	26
*Candida albicans*	10	13	16	20	24	—	25

**Table 4:** The minimum inhibitory concentration (MIC) of microbe's CSS NC in different concentrations (measured in *µ*g.ml-1).

Microorganism	Different concentrations of CSS NC (*µ*g.ml^−1^)									
	0.78	1.56	3.12	6.25	12.5	25	50	100	200	400
*Staphylococcus aureus*	+	+	+	+	−	−	−	−	−	−
*Bacillus cereus*	+	+	+	−	−	−	−	−	−	−
*Escherichia coli*	+	−	−	−	−	−	−	−	−	−
*Pseudomonas aeruginosa*	+	+	−	−	−	−	−	−	−	−
*Aspergillus niger*	+	−	−	−	−	−	−	−	−	−
*Candida albicans*	+	+	−	−	−	−	−	−	−	−

**Table 5:** The amount of phenolic compounds in the methanolic extract of *Pistacia vera L*. hull.

Phenolic compounds	The amount of compounds (mg/g dried material)
Gallic acid	55
Protocatechuic acid	0.39
Catechin	1.17
Rutin	132.6
Quercetin	2.3

**Table 6:** Structure of phenolic compounds identified in *Pistacia vera* L. hull.

Phenolic compounds	Structure
Gallic acid	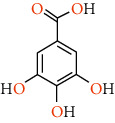
Protocatechuic acid	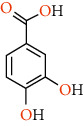
Catechin	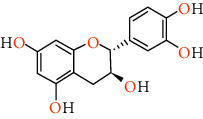
Rutin	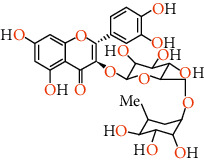
Quercetin	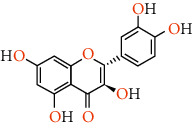

**Table 7:** Comparison of the amount of phenolic compounds (mg/g dried material) in *Pistacia vera* L. hull in this study (A) and in a similar study (B) [52].

Phenolic compounds	A	B
Gallic acid	55	5.7
Catechin	1.17	1.39
Quercetin	2.3	0.071

**Table 8:** Chemical composition of the essential oil from the *Pistacia vera* L. hull.

No.	Compounds	Percentage
1	Ethyl acetate	1.85
2	Tricyclene	1.13
3	*α*-Pinene	45.62
4	Camphene	7.76
5	*β*-Pinene	1.11
6	3-Caren	2.97
7	D-Limonene	12.16
8	Terpinolene	4
9	Limonene-4-ol	4.24
10	*α*-Terpineol	5.38
11	Isobornyl acetate	13.78
12	Total	100

**Table 9:** Chemical structure of *Pistacia vera* L. hull essential oil terpenes.

Terpene	Structure
Tricyclene	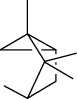
*α*-Pinene	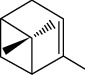
Camphene	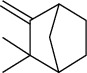
*β*-Pinene	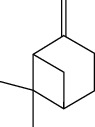
3-Caren	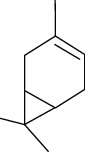
D-Limonene	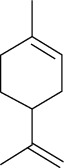
Terpinolene	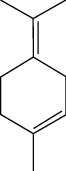
Limonene-4-ol	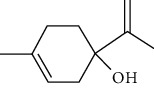
*α*-Terpineol	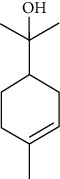
Isobornyl acetate	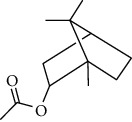

## Data Availability

The data used to support the findings of this study are available from the corresponding author upon request.

## Supplementary Materials

Supplementary Materials. Additional data can be found in this section.
