# Supplementary material for: Biosynthesis of Organic Nanocomposite Using Pistacia vera L. Hull: An Efficient Antimicrobial Agent
Source: Bioinorg Chem Appl. 2021 Jul 9;2021:4105853. doi: 10.1155/2021/4105853 (PMC8286193; doi:10.1155/2021/4105853)
Supplement: Supplementary Materials. — Additional data can be found in this section. [file 4105853.f1.docx]

**Biosynthesis of organic nanocomposite using *Pistacia vera* L. hull: An efficient antimicrobial agent**

Omolbanin Bakhshi, ^1^ Ghodsieh Bagherzade, ^1^ Pouya Ghamari kargar^1^

^1^ Department of Chemistry, Faculty of Sciences, University of Birjand, Birjand, 97175-615, Iran

Correspondence should be addressed to Ghodsieh. Bagherzade; bagherzadeh@birjand.ac.ir


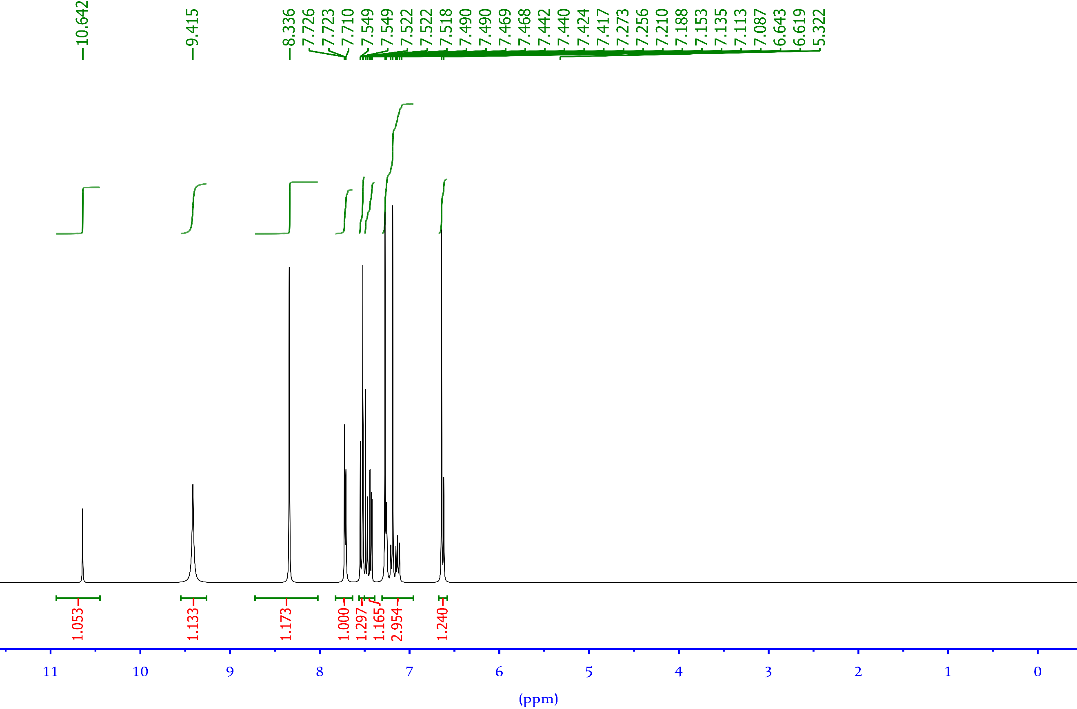


HNMR of SL


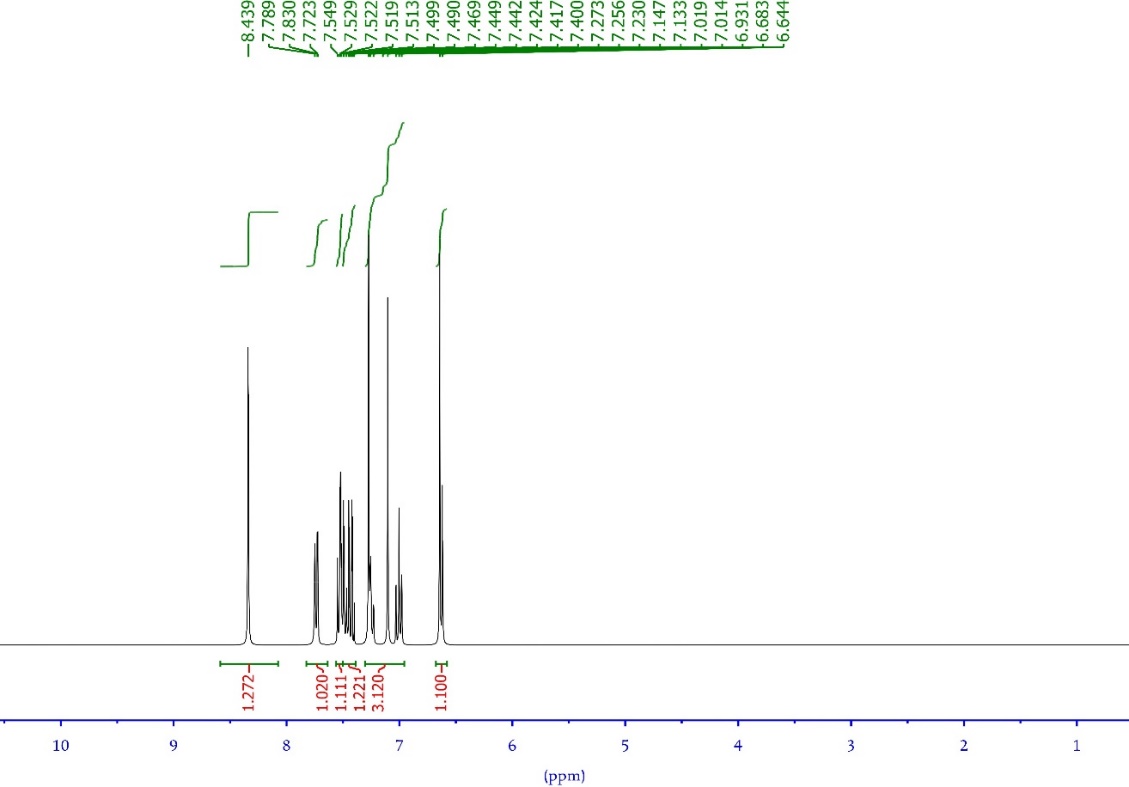


HNMR of CSS
